# Facile fabrication of carboxymethylcellulose/ZnO/g-C3N4 containing nutmeg extract with photocatalytic performance for infected wound healing

**DOI:** 10.1038/s41598-023-45921-7

**Published:** 2023-10-31

**Authors:** Maysa Youshi, Mohammad Reza Farahpour, Zohreh Ghazi Tabatabaei

**Affiliations:** 1 Department of Clinical Sciences, Faculty of Veterinary Medicine, Urmia Branch, Islamic Azad University, Urmia, Iran; 2 Department of Clinical Sciences, Faculty of Veterinary Medicine, Urmia Branch, Islamic Azad University, Urmia, Iran; 3 Department of Chemistry, Ahar Branch, Islamic Azad University, Ahar, Iran

**Keywords:** Biochemistry, Cell biology, Molecular biology, Plant sciences, Diseases, Health care, Chemistry, Materials science, Nanoscience and technology

## Abstract

New topical antibacterial agents are required to inhibit and development of bacteria and also promoting the wound healing process. This study was evaluating the healing effect of *Myristica fragrans* extract coated with carboxymethyl cellulose, zinc oxide and graphite carbon nitride (CMC/ZnO/g-C3N4/MyR) by photocatalytic process on the healing process of full-thickness infectious excision wounds in mice. Nanosheets were prepared and physicochemical properties were evaluated. Safety, in vitro release, antibacterial activities under in vitro and in vivo condition, wound contraction, histopathological properties and the protein expressions of tumor necrosis factor-α (TNF-α), collagen 1A (COL1A) and CD31 were also evaluated. Physicochemical properties confirmed their successful synthesis. Nanosheets exhibited antibacterial activity under in vitro and in vivo conditions. The formulations containing CMC/ZnO/g-C3N4/MyR, significantly (*P* < 0.05) competed with standard ointment of mupirocin for accelerating the wound healing process due to their effects on bacterial count and the expression of TNF-α and also accelerating the proliferative phase. This structure can be used as a safe structure in combination with other agents for accelerating the wound healing process following future clinical studies.

## Introduction

Wound infection is a common nosocomial infection that influences people all over the world and causes a high mortality rate^1^. Wound infection is due to bacterial pathogens entered into the body through the skin gaps such as *Pseudomonas aeruginosa, Staphylococcus aureus, Klebsiella pneumoniae* and *Acinetobacter baumannii*^2^. The preparation of a clean and moist wound environment that can support the physiological wound healing process is a managerial strategy for wound management^3^. Broad use of antibiotics results in drug resistance and the production of superbugs bacteria and can cause side effects on the immune system^4^. New topical antibacterial agents are required to inhibit the further spread and development of complications in fighting bacterial infections^5^. Medicinal plants have been used for accelerating infected wounds.

Nutmeg (*Myristica fragrans*) or MyR is a commercial source used as antithrombotic, antitumor, and anti-inflammatory^5^. It is a rich source of bioactive substances with antibacterial and antioxidant activities^6^. It is known to have the wound healing activities^7,8^. There are problems in dispersion of plant derivations and also drug delivery. It is essential to coating plant derivations with safe carriers with good water dispersibility such as nanosheets^9^. In addition, a combination of nanosheets and extracts can have better antimicrobial properties^10^.

The inorganic particles such as ZnO have antibacterial activities, antidiabetic and anticancer activities^11^. ZnO are economic and safe with optical and excellent thermal and chemical stability^12^. It is a candidate for the wound healing process due to its biological properties. In addition, a progressive opted strategy is producing hybrid nanostructures of ZnO via its coupling with other fascinating semiconducting materials. This work results in the preparation of structures with maximum biological and mechanical properties^13^. Graphitic carbon nitride (g-C3N4) is a metal-free photocatalyst that has attracted many attentions for the production of heterojunctions in the entire scientific society due to properties such as chemical and thermal stability, faster charge transport, and ability absorbing light^14^. It can be utilized as a photothermal agent for photothermal therapy^15^. Zn^2+^ doping could increase the photocatalytic activity of g-C3N4, and promote epithelial formation in the wound healing process^4^. Another agent which can be used for tissue engineering is carboxymethyl cellulose (CMC). The dressings based on natural polymers have been used for biomedical applications^16^. It has stability and biocompatibility properties^17^. It may have synergistic effects with g-C3N4 and Zn^2+^ for mechanical and biological properties in the treatment of wound.

It was hypothesized that CMC/ZnO/g-C3N4 can coat MyR and accelerate the wound healing process in the presence of visible light and have synergistic effects for the treatment of infected wounds. This study evaluates the effects of CMC/ZnO/g-C3N4/MyR on the treatment of infected wounds by assessing physicochemical properties, antibacterial activity and the expressions of tumor necrosis factor-α (TNF-α), angiogenesis (CD31) and collagen type 1A (COL1A).

## Results and discussion

g-C3N4 and metal oxides such as ZnO are the active semiconductors under visible light with a wide forbidden strip and can only be activated by ultraviolet light. When these particles absorb photons with higher energy than their banned strips, couples of electrons and cavities are created in the conduction bar and their capacity strips, respectively (Eq. 1).

1 $${\text{ZnO/g - C}}_{{3}} {\text{N}}_{{4}} + {\text{ h}}\nu \to {\text{ZnO/g - C}}_{{3}} {\text{N}}_{{4}} \left( {{\text{e}}^{ - }_{{{\text{CB}}}} + {\text{ h}}^{ + }_{{{\text{VB}}}} } \right)$$

High-efficiency separation in this couple will allow oxidation and reduction reactions on the particle surface. In these reactions, molecular oxygen commonly works as an electron acceptor and is converted into superoxide anion (O_2_^−⋅^ which is a strong oxidation agent, in fact e^−^ in CB (conduction bar) helps to produce superoxide radicals from oxygen molecules (Eq. 2).

2 $${\text{e}}^{ - }_{{{\text{CB}}}} + {\text{ O}}_{{2}} \to {\text{ O}}_{{2}}^{ - \cdot }$$

On the other hand, the cavity with surface hydroxyl group (OH^−^) with a water molecule (water in the hydrogel or wound bed) absorbed on the surface of the reaction produces a hydroxyl radical (OH°), which is a strong reducing agent. H + in VB (capacity bar) plays a key role in the production of hydroxyl radicals from H2O molecules or hydroxyl ions^18,19^ (Eq. 3)

3 $${\text{h}}^{ + }_{{{\text{VB}}}} + {\text{ H}}_{{2}} {\text{O}}/{\text{OH}}^{ - } \to {\text{ HO}}^{ \cdot } + {\text{ H}}^{ + }$$

Reactive oxygen species (ROS; HO^⋅^, O_2_^−⋅^) are a key component in photocatalytic process. On the other hand, the rapid recombination of the produced electron-cavitational, their photocatalytic activity has been accompanied by limitations. To improve the performance of such potential semiconductors, useful solutions have been taken to pair the two semiconductors together and place them on a suitable substrate^20,21^.

### GC–MS analysis of MyR extract

**Figure 1:**
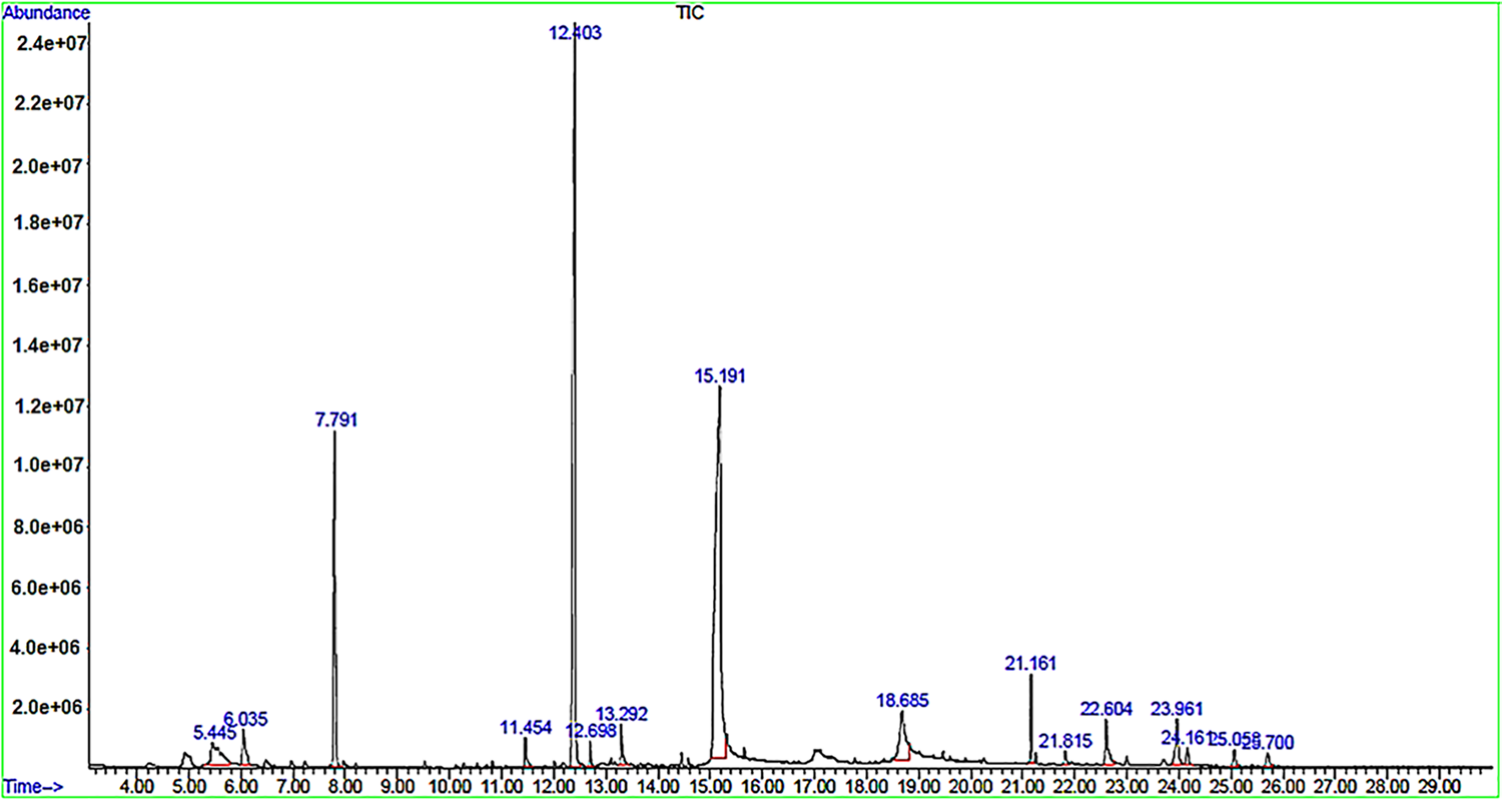
Chromatogram of *Myristica fragrans* (Nutmeg or MyR) extract.

The chemical composition of the extract was analyzed with the help of GC–MS technique. This technique (Fig. 1) identified and quantified 16 different compounds, representing 93% of the total extract (Table 1). Myristicin (32.17%), Myristic acid (25.81%), Terpinen-4-ol (11.99)%, and isooctyl phthalate (4.91%) were found as the main compounds that are in agreement with previous studies^22,23^. Other important compounds include 4-Terpinenyl acetate (3.81%), γ-Terpinene (1.84%), Isoeugenol (1.05%), Oleic acid (2.88%), Nomifensine (2.06%) and Carvacrol (2.25%). Differences in the chemical composition of nutmeg are due to differences in culture condition and solvent used for extraction^24^.

**Table 1:** GC/MS analysis of MyR extract.

No	Components	Retention time (min)	Amount (%)
1	4-Terpinenyl acetate	5.44	3.817
2	γ-Terpinene	6.03	1.846
3	**Terpinen-4-ol**	7.79	11.997
4	Isoeugenol	11.45	1.058
5	**Myristicin**	12.403	32.179
6	Elemicin	12.69	0.543
7	4-Allyl-2,6-dimethoxyphenol	13.29	1.185
8	**Myristic acid**	15.19	25.817
9	Oleic acid	18.68	2.889
10	**Isooctyl phthalate**	21.16	4.918
11	Benzeneacetaldehyde, 2,5 dimethoxy-α-methyl	21.81	0.485
12	Nomifensine	22.60	2.066
13	Carvacrol	23.96	2.259
14	Safrole	24.16	0.679
15	1,8-Cineole	25.05	0.660
16	Caryophyllene oxide	25.70	0.602
	Total	–	93.003

### Structural characterization

**Figure 2:**
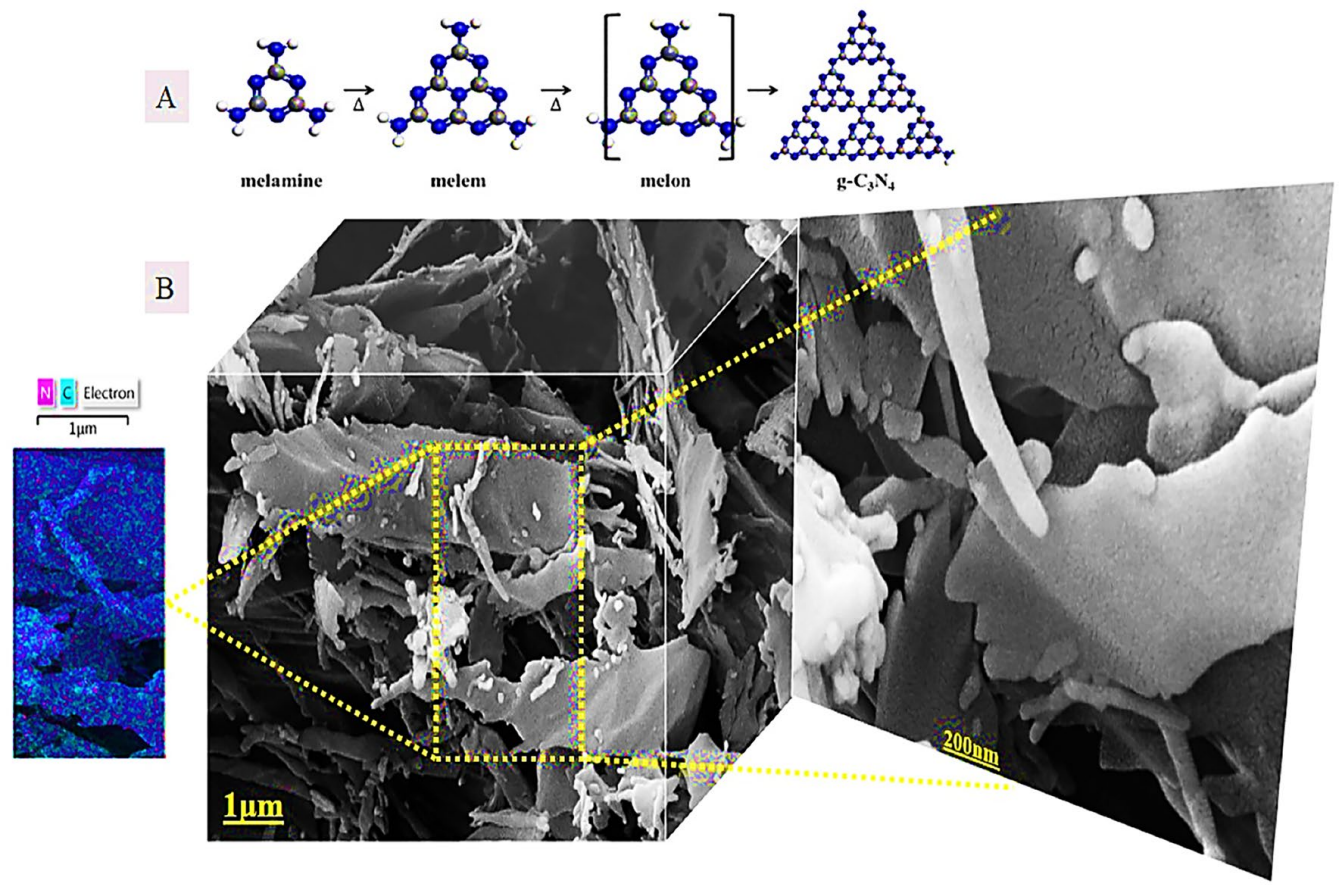
(**A**) Synthesis phases, (**B**) FESEM and dot mapping nanocrystals of g-C3N4.

**Figure 3:**
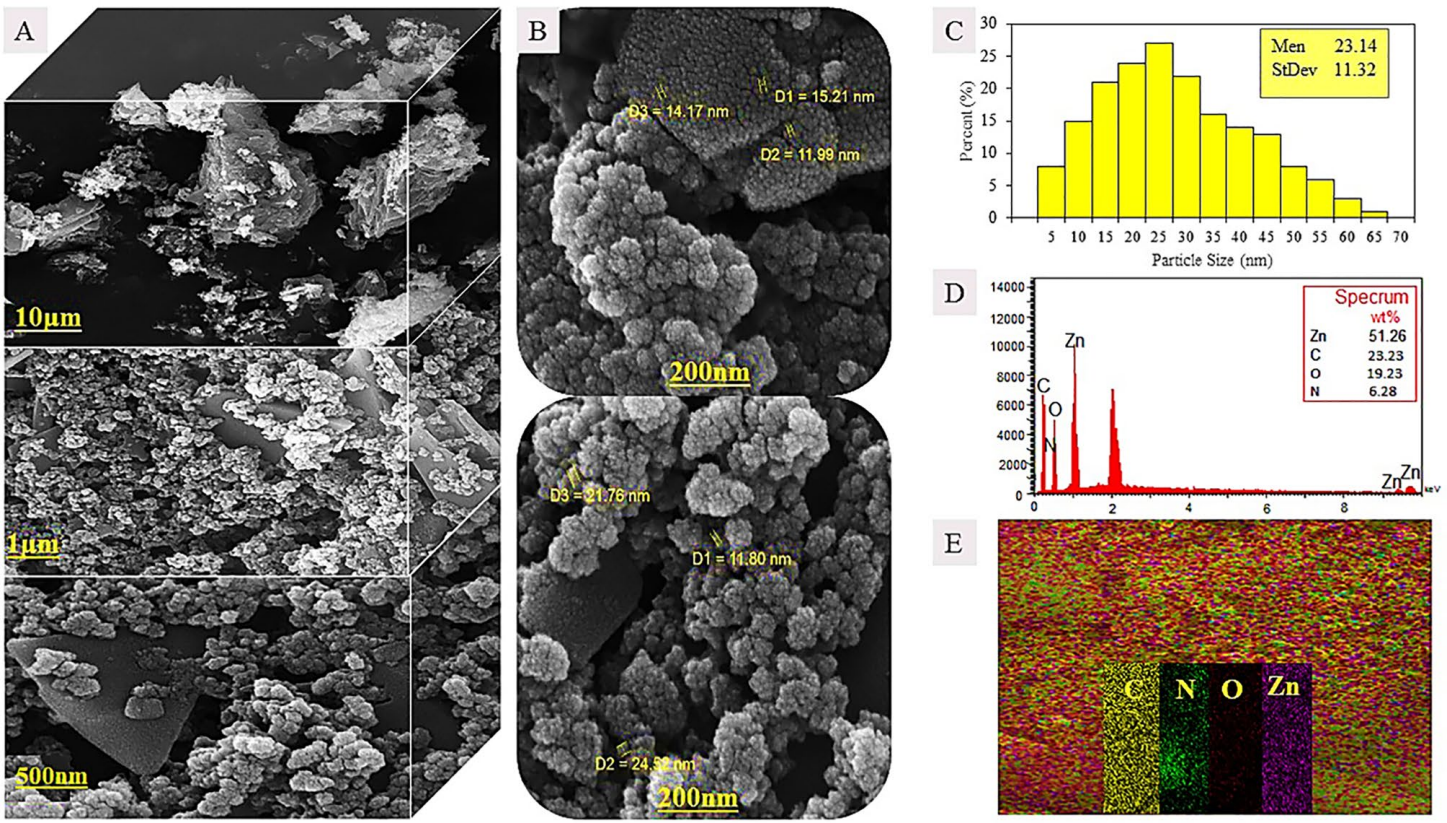
ZnO/g-C3N4 sample, FESEM (**A**) in different magnifications, (**B**) particle size and EDX, (**C**) PSD, (**D**) Dot mapping.

**Figure 4:**
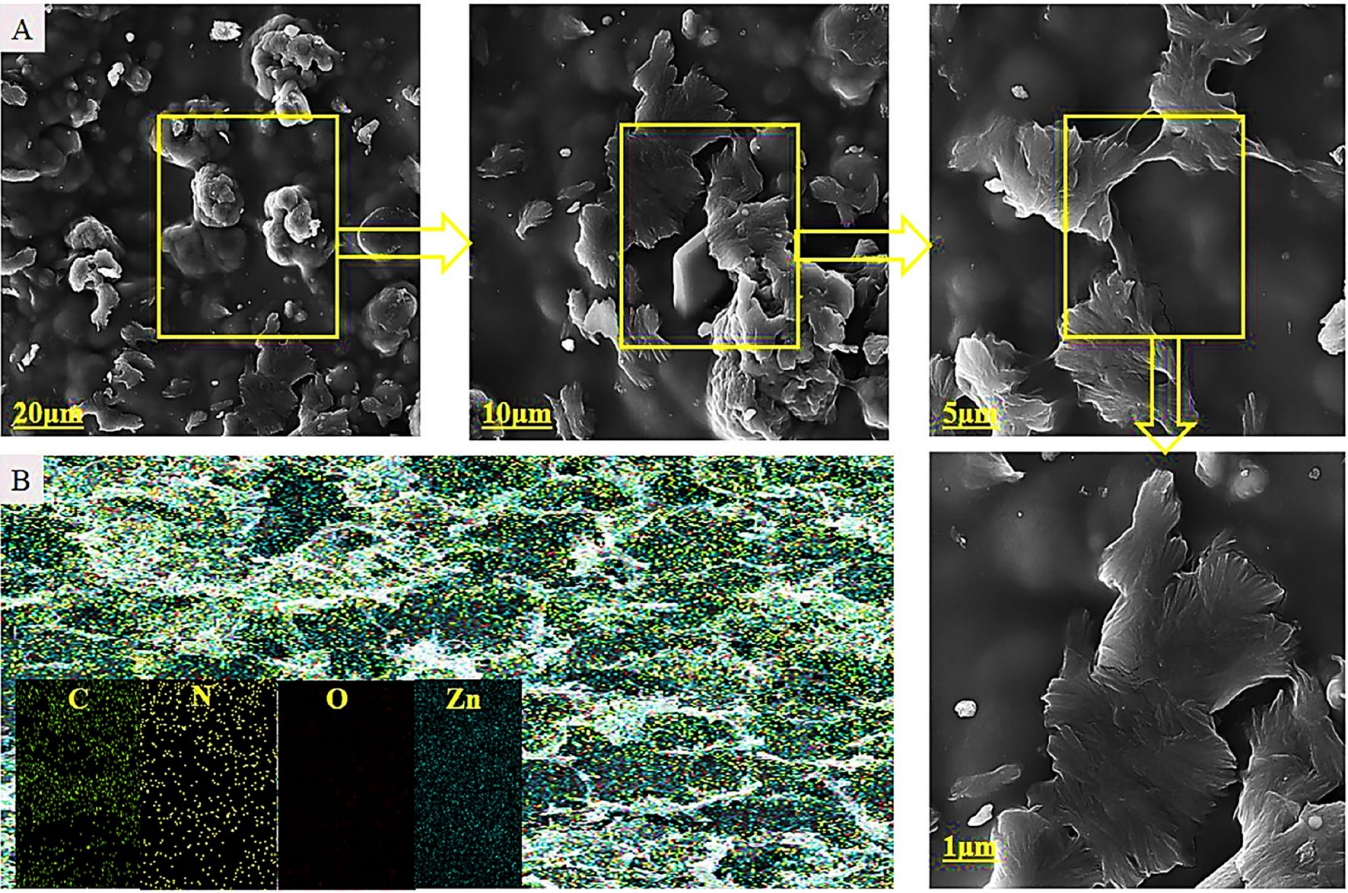
CMC/ZnO/g-C3N4 sample, FESEM (**A**) in different magnifications, (**B**) Dot mapping.

**Figure 5:**
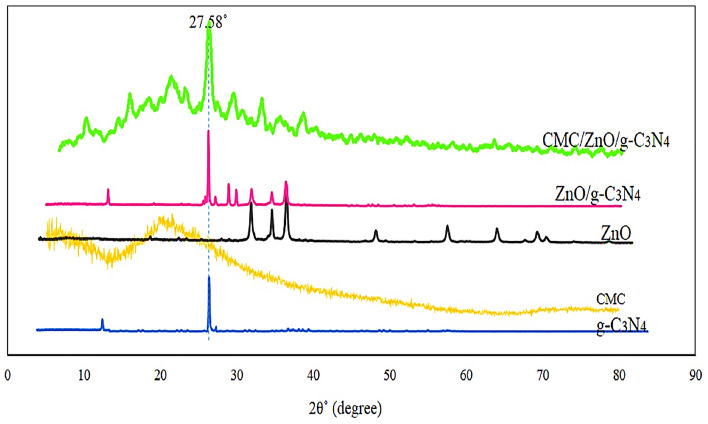
XRD patterns of g-C3N4, CMC, ZnO, CMC/ZnO/g-C3N4.

**Figure 6:**
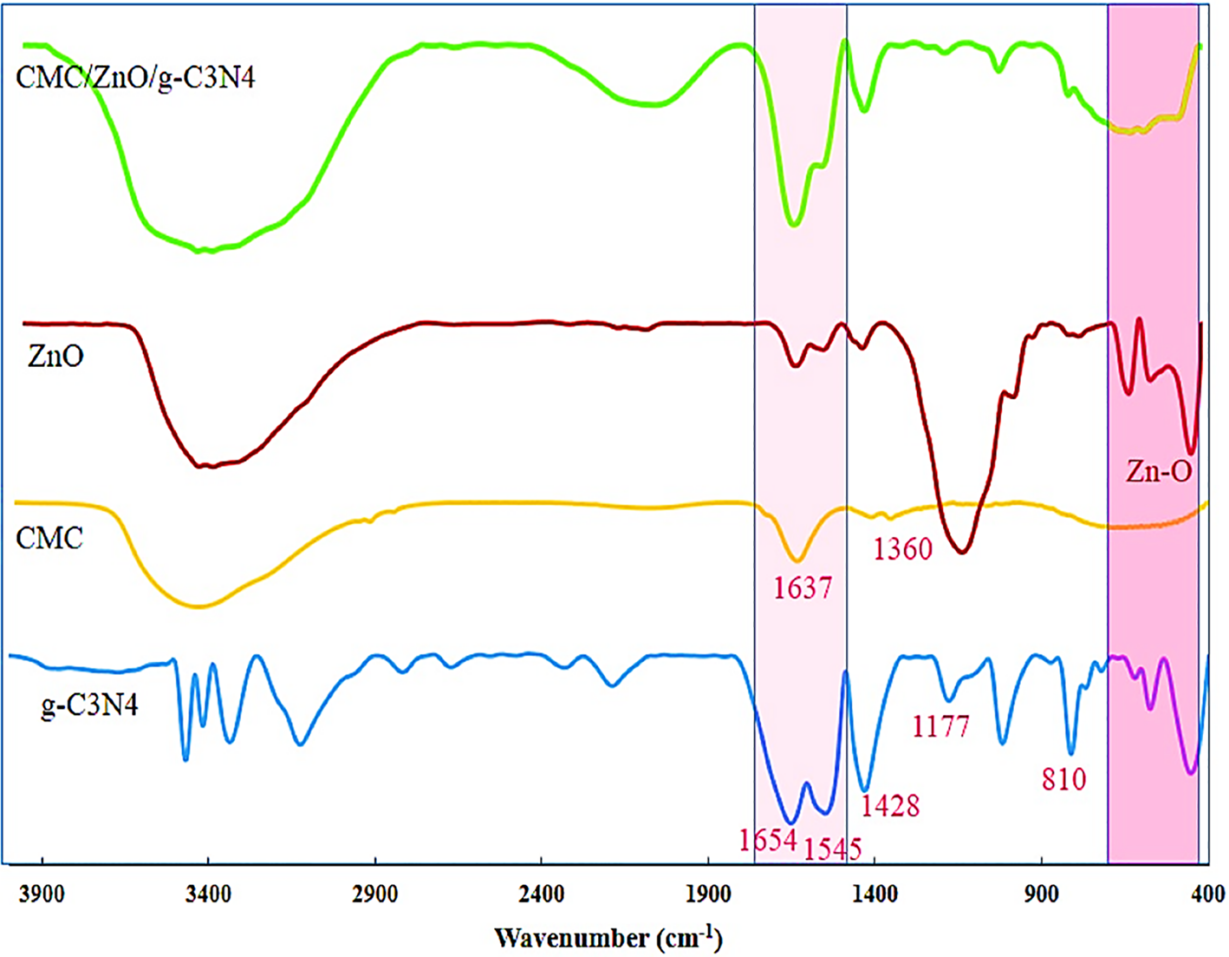
FTIR spectra for g-C3N4, CMC, ZnO and CMC/ZnO/g-C3N4.

To demonstrate the feasibility of this project, g-C3N4 was synthesized from melamine powder according to Fig. 2A and based on previous reports^25^. To obtain more g-C3N4 nanosheets, sonication was performed in water and the obtained g-C3N4 had several single layers based on the characteristics of electron-scanning microscopy (Fig. 2B). The g-C3N4 electron microscopy images show free standing nanosheets with a thickness size of 51 nm. Also, the distribution of C and N elements can be seen in dot mapping next to SEM. The g-C3N4 nanosheets were easily dispersible in water due to the presence of –NH2 and –NH groups which were confirmed by Fourier transform infrared spectroscopy (FTIR), it is shown in Fig. 6. Such groups –NH2 and -NH can act as an effective Lewis base for charging metal ions via chelate and have a higher binding affinity to ZnO (Fig. 3) and thus provide potential growth of nanoparticles at g-C3N4 level^26^. Accordingly, g-C3N4@ZnO a can be synthesized by a facile and simple reduction method in place at room temperature. SEM images (Fig. 3A,B) and histograms of the corresponding size distribution (Fig. 3C) showed that a medium density of dispersed ZnONP with an average size of 23 nm was precipitated on g-C3N4 surface and no accumulated ZnONP were observed. In addition, ZnONP modification on g-C3N4 surface was confirmed by energy dispersed X-ray spectroscopy (EDS) (Fig. 3D). Based on the EDS results obtained from ZnO/g-C3N4 sample, 23.23% carbon, 6.28% nitrogen, 51.26% Zn and 19.23% oxygen were found. Dot mapping results (Fig. 3E) showed a uniform dispersion of elements on the surface of nanosheets. ZnO/g-C3N4 is located on CMC substrate, and SEM images (Fig. 4A) and dot mapping (Fig. 4B) are shown that the proper and uniform distribution of elements (C, N, O, and Zn) in CMC substrate is visible. XRD patterns were used to identify the crystalline structure of the materials (Fig. 5). The index peak for g-C3N4 sample at 13.49 °C is related to tri-s-triazine units and intra-plate structural accumulation and is dedicated to the plate (100). The peak of the index is 27.58 °C to the plate (002) and shows that the conjugated aromatic groups of g-C3N4 layers have appeared with the JCPDS No reference card (87–1526)^27^. For example, ZnO index peaks were 32.08°, 34.5°, 36.31°, 47.78°, 56.80°, 63.07° and 68.31° which correspond to crystalline plates Hexagonal structure (100), (002), (101), (102), (110), (103) and (112) and were in accordance with reference card (JCPDS No. 36-1451)^28^. For the broad peak polymer matrix in the range of 20.4, the amorphous state of CMC is well represented. In ZnO/g-C3N4 and CMC/ZnO/g-C3N4 peaks of crystalline index were visible in ZnO and g-C3N4 that confirmed the synthesis of the final product (Eq. 4).

4 $$d = \frac{k\lambda }{{\beta \cos \theta }}$$

In this equation, d is the average particle size (nm), k = 0.89, 0.154060 = λ nm for Cu Ka and β full width is half the peak maximum. Using the Debye-Scherr equation, the mean crystal size of ZnO/g-C3N4 nanoparticles and CMC/ZnO/g-C3N4 were 26.14 and 77.43 nm, respectively, which was consistent with other analyses such as SEM, DLS and PSD. The FTIR spectrum of g-C3N4, CMC, ZnO and CMC/ZnO/g-C3N4 sheets is shown in Fig. 6. The pure FTIR spectrum of g-C3N4 has a wide absorption band from 3000 to 3400, which shows tensile states (NH-) and (NH2-). Peaks at 1177 cm^−1^, 1428 cm^−1^, 1545 cm^−1^ and 1654 cm^−1^ are related to bonds (C-N) and (C=N). In addition, the peak shown in 810 cm^−1^ is related to the ES-triazine ring units^29^. In the CMC peak spectrum appeared at 3600–13,400 cm^−1^ is associated with the stretch vibrations of aromatic ring hydroxyl groups and the peaks that appeared in 1360 cm^−1^ are related to the asymmetric tensile vibrations of methyl (–CH) carboxymethyl cellulose groups. The asymmetric tensile vibration of the COO group closed to 1637 cm^−1^ and vibration at 1093 cm^−1^ was assigned to C–O tensile vibration. Zn–O extended band was seen in the range of 560–430 cm^−1^^30^. It can be seen that all peaks in the first three spectrums are visible in the fourth peak, indicating the successful synthesis of the CMC/ZnO/g-C3N4 composite.

**Figure 7:**
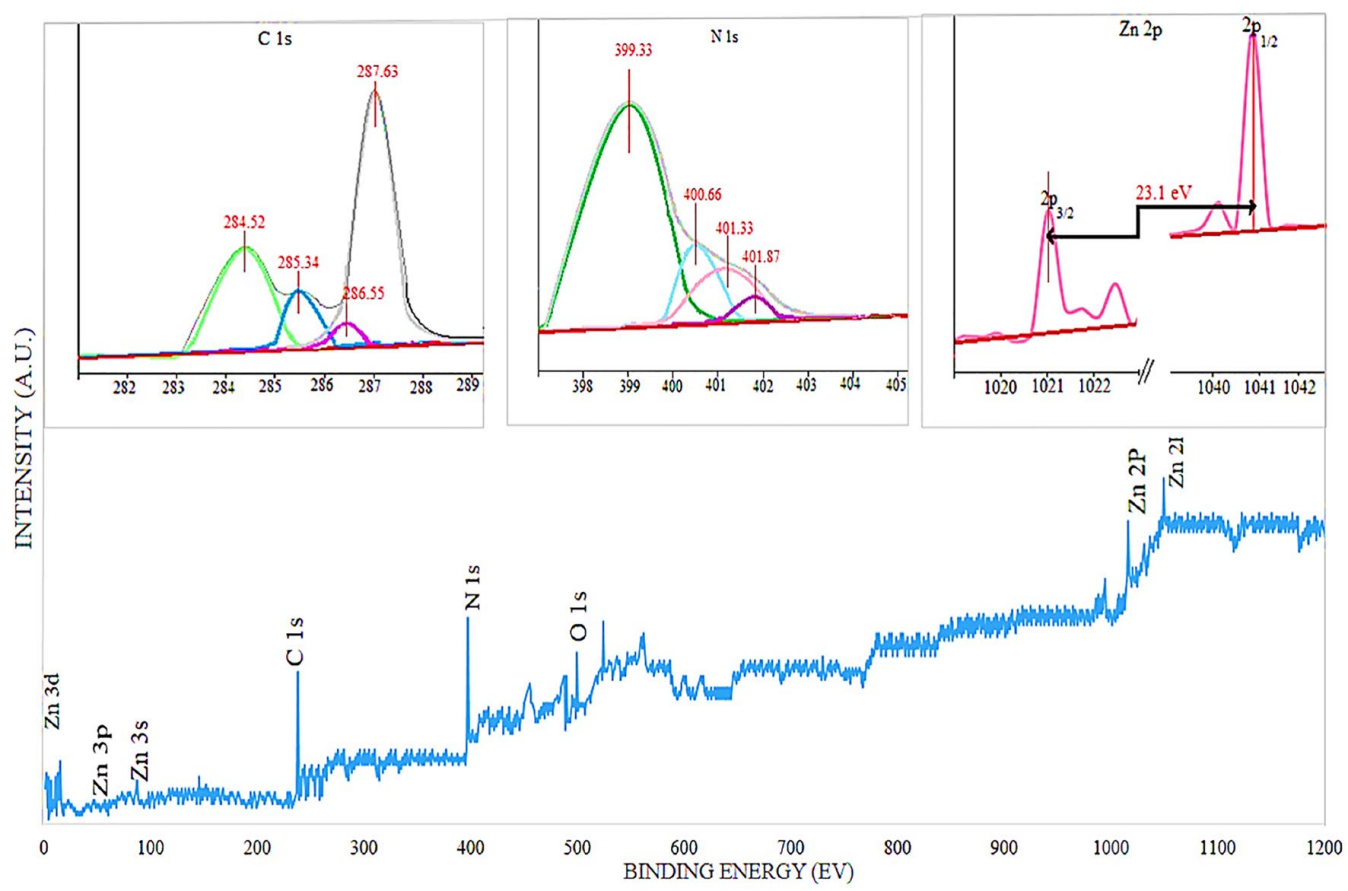
XPS spectra of CMC/ZnO/g-C3N4.

The chemical composition and oxidation state of the elements were determined by XPS. As shown in Fig. 7, the wide range of XPS, CMC/ZnO/g-C3N4 shows that the index peaks are related to C, N, O and Zn. As the XPS C1s spectrum shows in Fig. 7. Four peaks are observed at 284.52 eV, 285.34 eV, 286.55 eV and 287.63 eV, which are attributed to C–C/C=C, C–N/C–O, C=N/C=O and O=C–O. Spectrum N 1 s was converted to four peaks at 399.33, 400.66, 401.33 and 401.87 eV, which is dedicated to Pyridine-N, pyloric-N, graphitic-N and N-Ox^31^. Typical Zn 2p peaks at 1021.6 eV and 1044.7 eV are attributed to Zn 2p1 and Zn 2p3 respectively, which are in agreement with the normal Zn binding energy in ZnO. In addition, the O 1 s spectrum is characterized by two specific peaks, 229.9 eV and 351.6 eV are related to Zn–O and N–C–O, respectively. XPS results showed that nanosheet was successfully constructed without any impurities.

**Figure 8:**
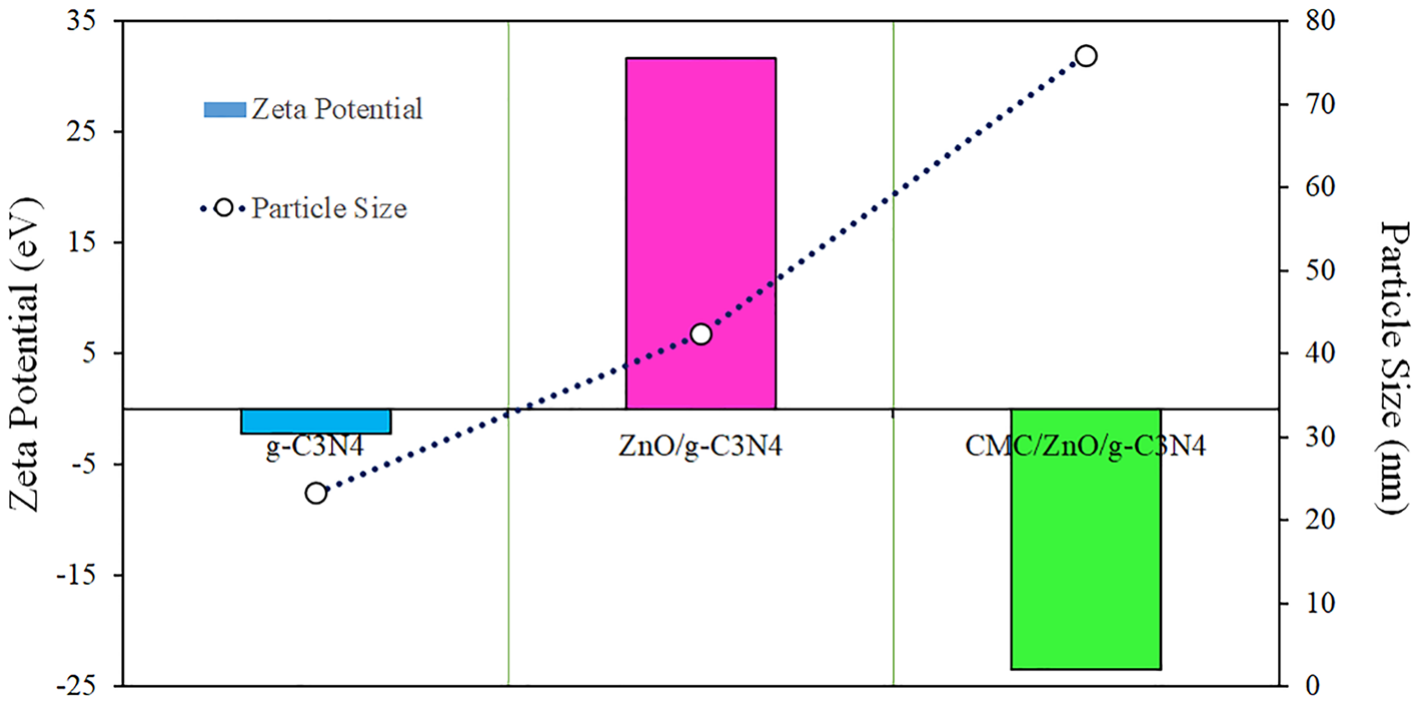
DLS and zeta potential of g-C3N4, ZnO/g-C3N4 and CMC/ZnO/g-C3N4.

Particle size and zeta potential values for g-C3N4, ZnO/g-C3N4 and CMC/ZnO/g-C3N4 nano sheets are shown in Fig. 8 diagram (Fig. S1). Zeta potential plays an important role in the physical stability of particles^17^. Higher values of zeta potential (positive and negative) indicate its stability for nanoparticles. Zeta potential for g-C3N4 is negative (− 2.3 mV), and adding ZnO results in value positive (+ 29.1 mV). Decorating the surface of the CMC/ZnO/g-C3N4 nano sheets changed the zeta potential from positive to negative (− 23.4 mV) that is in agreement with previous studies^32,33^.

**Figure 9:**
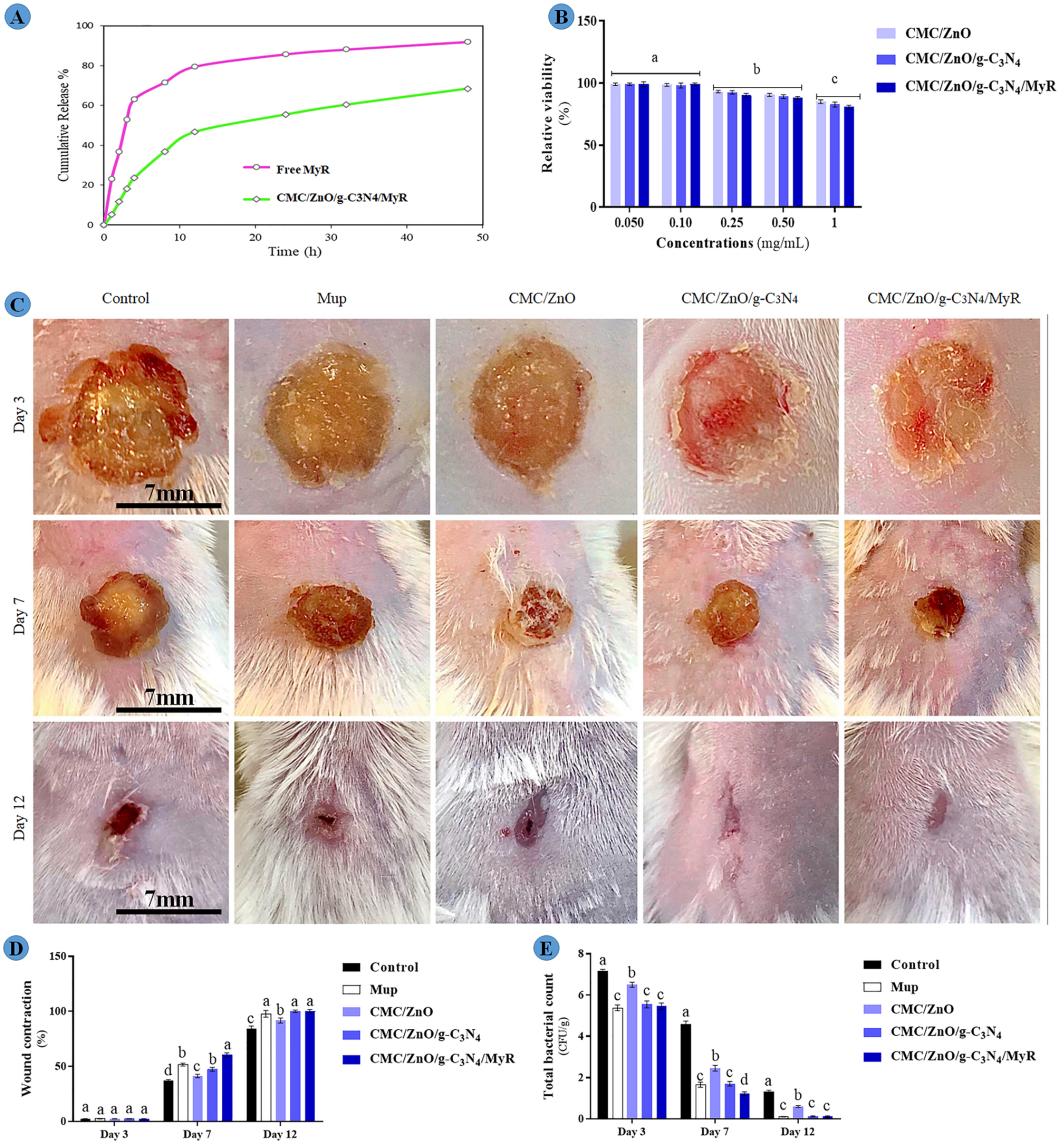
(**A**) Release profile of MyR and CMC/ZnO/g-C3N4/MyR; (**B**) cytotoxicity of nanosheets; (**C**) gross wound image; (**D**) wound contraction percentage, and (**E**) total tissue bacterial count in experimental groups on different days. Data are expressed as mean ± standard deviation (n = 6). Different letters (a-d) show significant differences (*P* < 0.05) between groups.

Figure 9A shows the in vitro release of free MyR and CMC/ZnO/g-C3N/MyR over 48 h. Considering that during the wound healing process, the pH of the wound area changes dynamically from pH 4 to pH 8, for growth factor secretion and activation^34,35^. The underlying wound tissue shows a neutral pH of 7.4. As a result, the release profile of CMC/ZnO/g-C3N4 containing MyR at pH 7.4 was investigated. In vitro release was significantly higher in free MyR compared to CMC/ZnO/g-C3N/MyR. During the first 4 h, 63.17% of MyR was released from MyR solution in the buffering environment while CMC/ZnO/g-C3N4/MyR about 23.63% is released in the first 4 h. Over time up to 8 h, the released values were 91.92%, 60.37% of dialysis bags for free MyR, CMC/ZnO/g-C3N4/MyR respectively. The possible reason is the complex polymer matrix network containing CMC/ZnO/g-C3N4, which releases it slowly. Since this matrix is also considerably resistant to degradation, the emission of the extract from the polymer matrix takes a longer time. With the slow release, probably due to the decrease in MyR concentration in healthy tissues and reduction of the toxicity of the extract, the coated form of nutmeg can have equal effects on the free drug in the treatment of infections and wounds, and polymeric coating can be used to control the release of the extract.

### Cytotoxicity

Figure 9B illustrates the results for the cytotoxicity of nanosheets. The results showed that nanosheets had lower toxicity. The highest toxicity was observed in the highest concentration (85.00%) for all the nanosheets. The different concentrations did not show significant differences. The findings are in accordance with literature for the safety of CMC^36^, ZnO^37^, g-C3N4^38^ and MyR^39^. A combination of materials could not have significant cytotoxicity. Toxicity was mostly associated with higher concentrations but not combinations of materials.

### Antibacterial activity

Table 2 shows in vitro antibacterial of the nanosheets in MIC and MBC tests. The results compared the effects of nanosheets compared with commercial agents. The results showed lowest antibacterial activities in MIC and MBC tests were observed in CMC/ZnO nanosheets. The addition of g-C3N4 could increase antibacterial activity for both bacteria. The addition of MyR could improve antibacterial activity compared with CMC/ZnO/g-C3N4. The results did not show significant differences between commercial antibiotics with CMC/ZnO/g-C3N4/MyR nanosheet and commercial antibiotics in MIC test. However, CMC/ZnO/g-C3N4/MyR nanosheet exhibited higher antibacterial activity compared with commercial antibiotics. The results are in accordance with previous studies for the antibacterial activity of CMC and ZnO^40,41^. Antibacterial activities of CMC/ZnO could be attributed to their interactions with reactive oxygen species and also the interaction of zinc oxide with bacterial cells wall and intra-cellular content of the cell such as protein, lipid, and carbohydrates which result in disruption of nucleic acids and bacteria death^42^. The addition of g-C3N4 increased antibacterial activity in nanosheets. The results are in agreement with other studies on the antibacterial activities of g-C3N4/ZnO^4^. The mechanism of g-C3N4/ZnO is attributed to their effects on disrupting membranes and increasing protein leakage, induction of bacterial apoptosis, and decreasing ATP levels^4^. The addition of MyR increased antibacterial activities. Major compounds of MyR were including 4-Terpinenyl acetate, γ–Terpinene, isoeugenol, oleic acid, nomifensine and carvacrol which disrupt bacterial membranes^43^. The results show synergistic effects between compounds for antibacterial activities. The CMC/ZnO/g-C3N4/MyR nanosheet could compete with commercial ointments.

**Table 2:** In vitro antibacterial of the nanosheets in MIC and MBC tests (µg/mL).

Agents	MIC	
	*Pseudomonas aeruginosa*	*Staphylococcus aureus*
CMC/ZnO	1.25	0.625
CMC/ZnO/g-C3N4	0.625	0.312
CMC/ZnO/g-C3N4/MyR	0.312	0.156
Meropenem	0.312	–
Mupirocin	–	0.156
P-value	0.01	0.01

Agents	MBC	
	*Pseudomonas aeruginosa*	*Staphylococcus aureus*
CMC/ZnO	2.50	1.25
CMC/ZnO/g-C3N4	1.25	0.625
CMC/ZnO/g-C3N4/MyR	0.625	0.312
Meropenem	1.25	–
Mupirocin	–	0.625
P-value	0.01	0. 01

### Wound size and tissue bacteria

Figure 9C,D shows wound healing activity of nanosheets on different days. The results did not show significant differences between groups on day 3 (P = 0.914). The highest wound healing activity were observed in the mice treated with formulations prepared from CMC/ZnO/g-C3N4/MyR on days 7 (P = 0.001) and 12 (P = 0.001) compared with other groups. The values for wound contraction were 37.16 ± 0.90%, 51.60 ± 1.30%, 41.28 ± 1.60%, 47.33 ± 1.80% and 60.50 ± 1.90% in control, Mup, CMC/ZnO, CMC/ZnO/g-C_3_N_4_ and CMC/ZnO/g-C_3_N_4_/MyR, respectively on day 7. The values for wound contraction were 84.21 ± 2.50%, 97.75 ± 2.10%, 91.62 ± 2.30%, 99.25 ± 0.85% and 99.56 ± 0.2% in control, Mup, CMC/ZnO, CMC/ZnO/g-C_3_N_4_ and CMC/ZnO/g-C_3_N_4_/MyR, respectively on day 12. There were no significant differences between those treated with CMC/ZnO/g-C3N4 and mupirocin on days 7 and 12 (P > 0.05); however, these exhibited lower wound contraction compared with those treated with CMC/ZnO/g-C3N4/MyR. Wound contraction was lower in those treated with CMC/ZnO compared with those treated with nanosheets (*P* < 0.05). The lowest wound contraction was observed in control mice on days 7 and 12. The results are in agreement with other studies for wound healing activities of CMC^44^, ZnO^45^, g-C3N4^4^ and MyR^8^. The results show synergistic effects between compounds for the wound healing process. The wound healing process of nanosheets could be attributed to their effects on bacteria and also the expression of genes. The nanosheets shorten the inflammatory phase and promote the wound healing process, as will be seen.

Figure 9E illustrates the photocatalytic effects of nanosheets on total bacterial count on different days. The highest total bacterial count was observed in the control mice compared with other mice (P = 0.001). The mice treated with CMC/ZnO showed lower total bacterial count compared with those in control group in all the days (P = 0.001) but the same mice showed higher total bacterial count compared with those treated with mupirocin and other nanosheets on all the days. The addition of g-C3N4 and MyR could decrease total bacterial count and the lowest total bacterial count was seen in the mice treated with CMC/ZnO/g-C3N4/MyR on day 3 and 7. The values for total bacterial count were 7.15 ± 0.10 CFU/g, 5.38 ± 0.14 CFU/g, 6.50 ± 0.12 CFU/g, 5.55 ± 0.17 CFU/g and 5.46 ± 0.15 CFU/g in control, Mup, CMC/ZnO, CMC/ZnO/g-C_3_N_4_ and CMC/ZnO/g-C_3_N_4_/MyR, respectively on day 3. The values were 4.58 ± 0.16 CFU/g, 1.65 ± 0.13 CFU/g, 2.45 ± 0.14 CFU/g, 1.70 ± 0.11 CFU/g and 1.21 ± 0.10 CFU/g in control, Mup, CMC/ZnO, CMC/ZnO/g-C_3_N_4_ and CMC/ZnO/g-C_3_N_4_/MyR, respectively on day 7. The values were 1.30 ± 0.08 CFU/g, 0.1 ± 0.02 CFU/g, 0.6 ± 0.04 CFU/g, 0.12 ± 0.03 CFU/g and 0.11 ± 0.04 CFU/g in control, Mup, CMC/ZnO, CMC/ZnO/g-C_3_N_4_ and CMC/ZnO/g-C_3_N_4_/MyR, respectively on day 12. It shows that CMC/ZnO/g-C3N4/MyR exhibits their effects on bacteria on first days. The mechanism of action of nanosheets was previously discussed under in vitro section. There is a good agreement between antibacterial results in both sections.

### Histopathological evaluation

**Figure 10:**
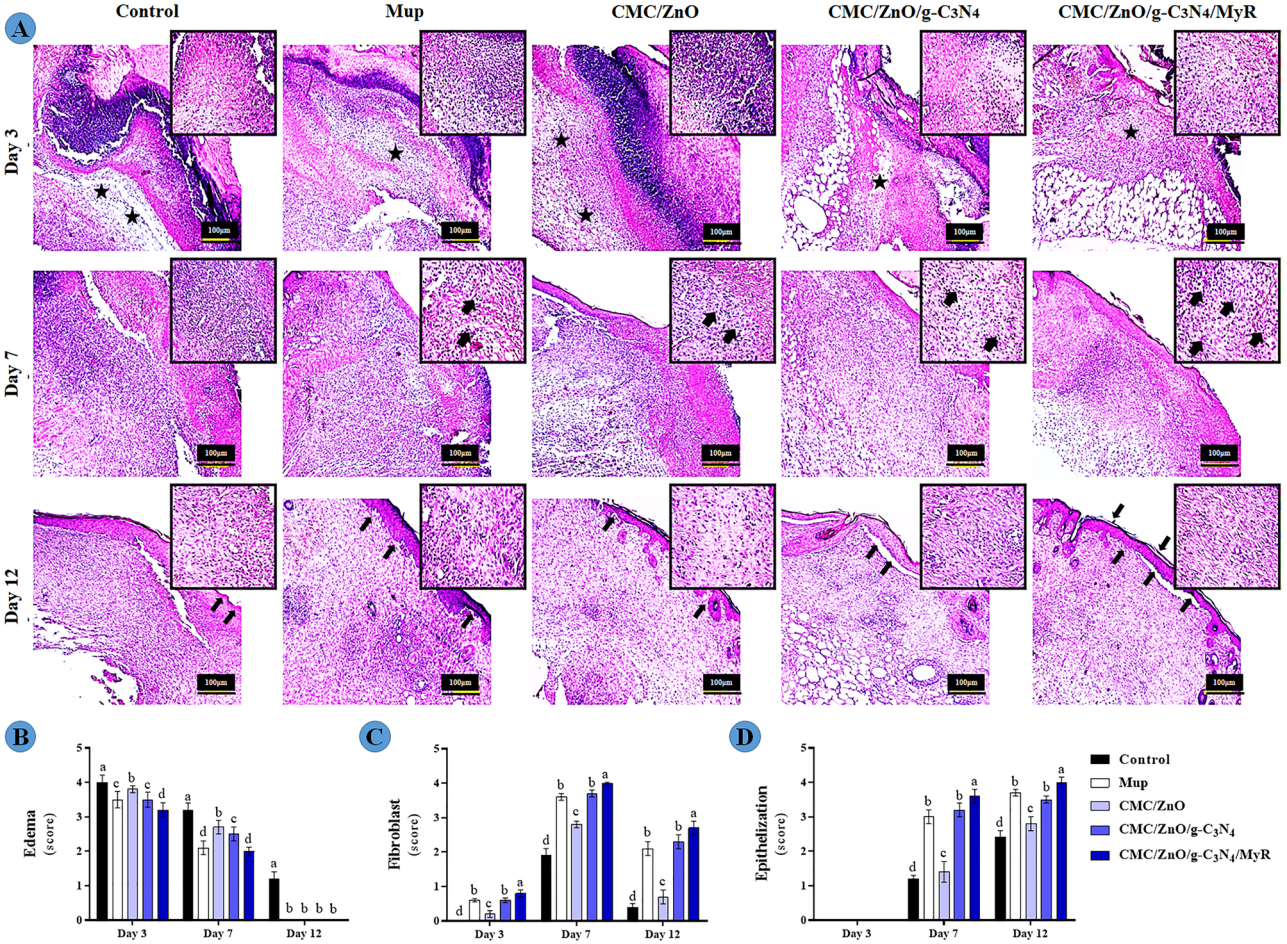
(**A**) The hematoxylin and eosin staining (**a**) of wounded samples of experimental groups. Note: Stars show edema intensity, arrows on day 7 show fibroblast cells and on day 12 epithelial thickness. An interesting point is that in the CMC/ZnO/g-C3N4/MyR group, the stratum corneum layer, unlike the other groups, has been formed; (**C**,**D**) the determined edema, fibroblast and epithelium thickness were scored as 0: absence;1: small amount; 2: low; 3: high and 4: severe at days of 3, 7, and 12. Non-similar letters on figures (**a**–**d**) show significant differences (*P* < 0.05) between groups.

The results for the effects of nanosheets on histopathological parameters are illustrated in Fig. 10. The results showed that edema was significantly higher in the control mice compared with other mice on days 3–12. The addition of g-C3N4 and MyR could decrease edema and the lowest total bacterial count was seen in the mice treated with CMC/ZnO/g-C3N4/MyR on all the days. The lowest fibroblast, and re-epithelization were observed in the control mice. The treatment of mice with commercial ointment and formulations prepared from CMC/ZnO/g-C3N4/MyR and CMC/ZnO/g-C3N4 increased fibroblast, and re-epithelization. The mechanism of action of nanosheets on pathological parameters could be attributed to their photocatalytic effects on the expression of genes, as discussed. Seemingly, nanosheets shift the wound healing process from the inflammatory phase toward proliferative phase and expedite the wound healing process.

### Immunofluorescent staining

**Figure 11:**
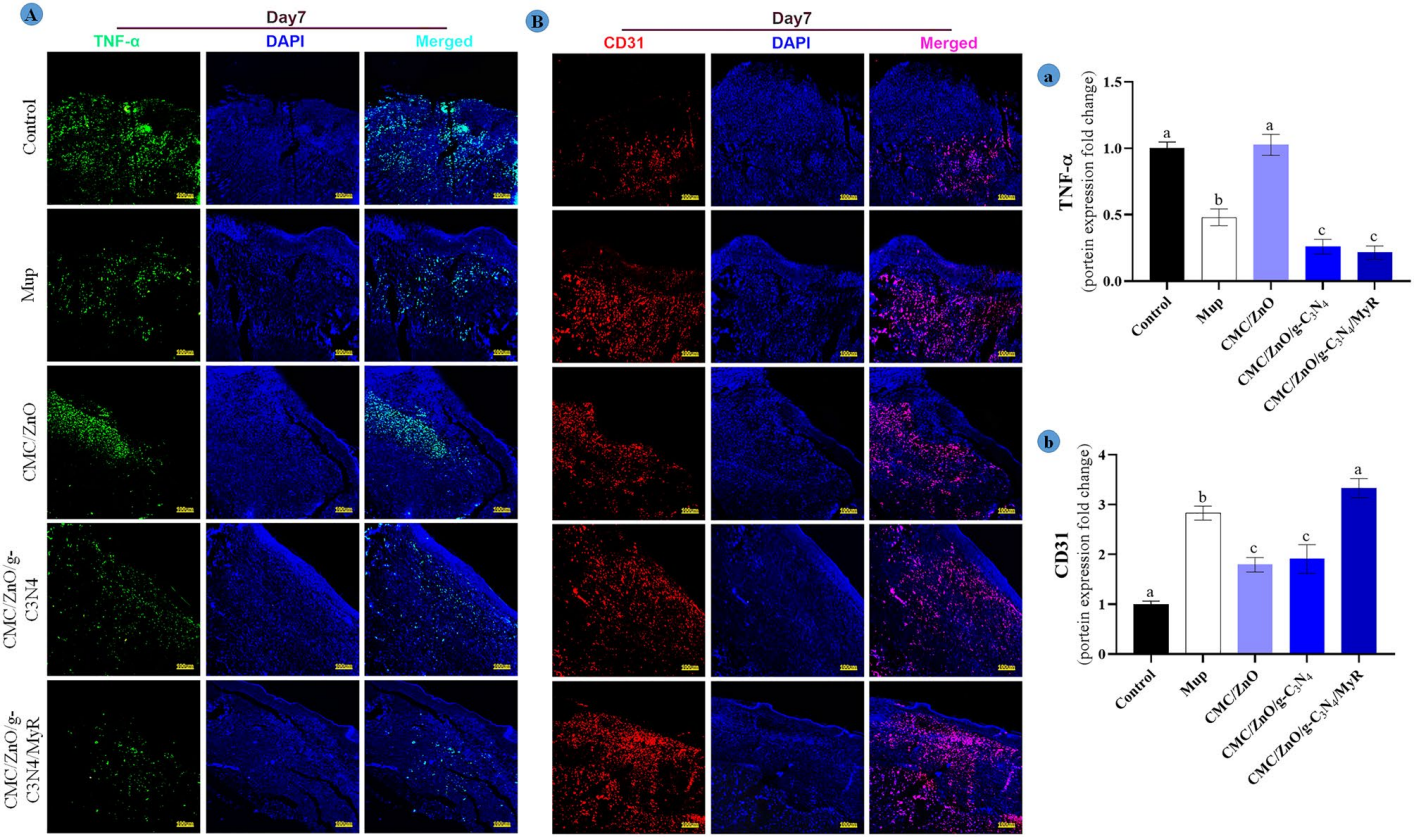
(**A**) Immunofluorescence staining for expression of TNF-α (green area) and CD31 (red area); and (**a**,**b**) quantitative statistics based on TNF-α and CD31 staining on day 7 at wound sites, respectively. Non-similar letters on figures (**a**–**d**) show significant differences (*P* < 0.05) between groups.

**Figure 12:**
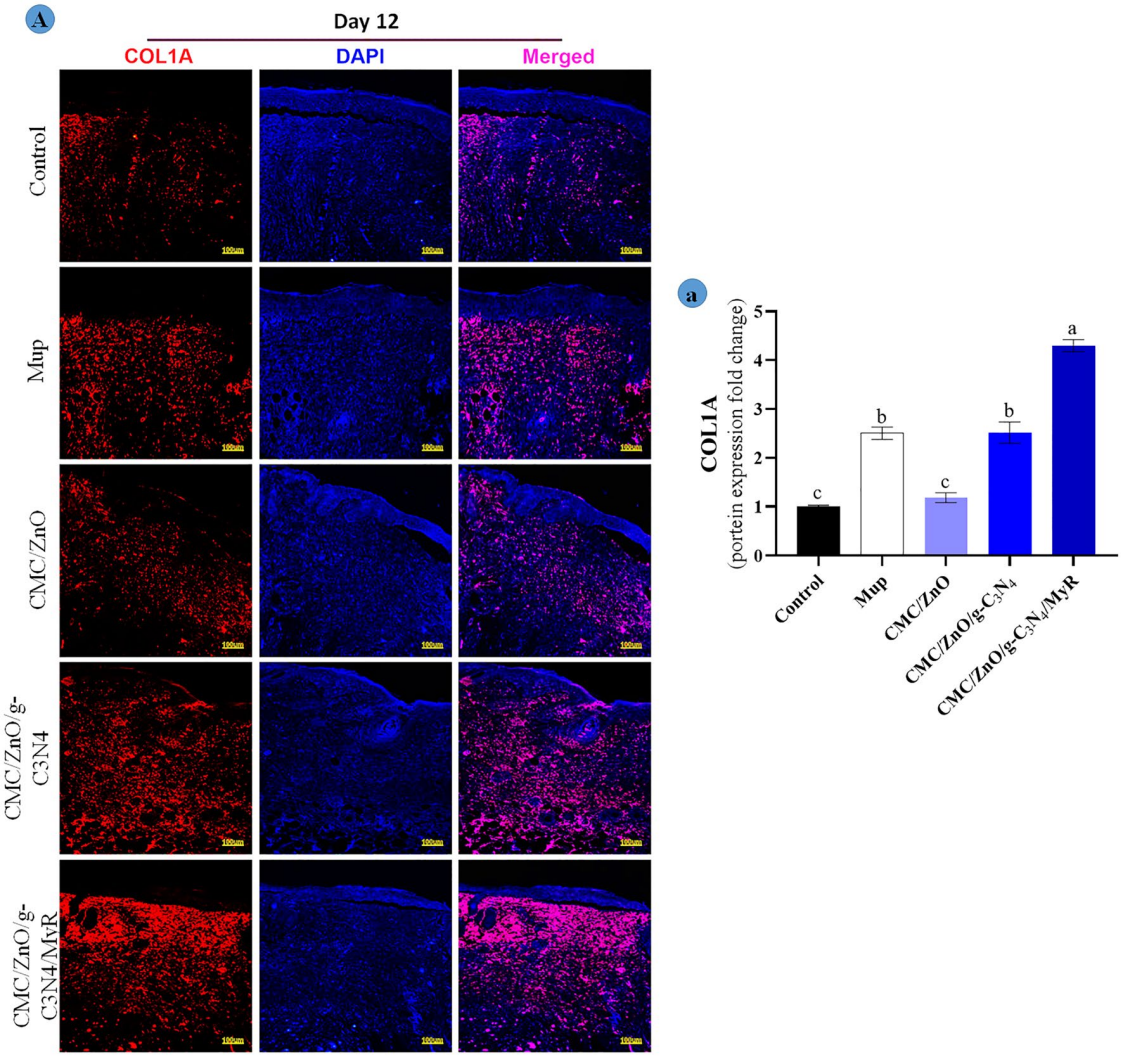
(**A**) Immunofluorescence staining for expression of COL1A (red area); and (**a**) quantitative statistics based on COL1A staining on day 12 at wound sites, respectively. Non-similar letters on figures (**a**–**d**) show significant differences (*P* < 0.05) between groups.

The results for the effects of synthetized nanosheets on TNF-α, CD31 and COL1A protein expression with immunofluorescence staining technique are shown in Figs. 11 and 12. The results showed that protein expression of CD31 and COL1A markers were significantly (*P* < 0.05) higher in the mice treated with CMC/ZnO/g-C3N4/MyR nanosheet formulation. The mice in control group showed lower the protein expression for CD31 and COL1A markers compared with other groups. The mice treated with mupirocin and CMC/ZnO/g-C3N4 had higher for collagen and CD31 compared with those treated with CMC/ZnO. The mice treated with CMC/ZnO/g-C3N4 and CMC/ZnO/g-C3N4/MyR showed significantly (*P* < 0.05) lower expression of protein for TNF-α compared with other groups. There were no significant differences between control mice and CMC/ZnO for the expression of TNF-α. Collagen plays a major role in contracting the wound healing process. The increase in the expression of collagen is due to the effects of nanosheets on the fibroblast proliferation. Previous studies indicated on M2 macrophages increase the fibroblasts migration and production of collagen 1 and 3^46^ which results in wound closure and accelerating wound healing process. In other hand, TNF-α is associated with M1 macrophage and delays wound healing processes^47^. The decrease in the expression of TNF-α and the increase in the expression of collagen promote the wound healing process by the treatment with nanosheets. The results show that nanosheets promote the wound healing process by contracting wound and decreasing the inflammation. In addition, CD31 marker participates in production of vessels (angiogenesis) and helps to supply nutrients for the wound healing process^48^. In sum, different compounds have synergism interaction effects for decreasing the inflammation and accelerating the wound healing process via angiogenesis and mature collagen synthesis.

## Conclusions

In this study, CMC/ZnO/g-C3N4/MyR quaternary biocomposites were successfully synthesized and physicochemical properties confirmed their properties. The results confirmed their safety and antibacterial activities under in vitro and in vivo conditions. This study showed that CMC/ZnO/g-C3N4/MyR nanosheets with photocatalytic performance could significantly expedite the infected wound healing process by decreasing total bacterial count, edema and the expression of TNF-α and also increasing angiogenesis, mature collagen synthesis and epithelization. CMC/ZnO/g-C3N4/MyR nanosheets can compete with standard ointment of mupirocin. It can be used in combination with other ointments for the treatment of clinical wounds following future studies. Novelty is a strength point for this study and murine study is a major limitation that cannot be used for clinical uses in humans.

## Materials and methods

### Materials

Melamine (C3H4N6, 99%), methanol (ACS grade, 99%), and phosphate citrate buffer were prepared from Sigma Aldrich Company (Chemie, Steinheim, Germany). Zinc acetate dihydrate with 99.9% purity, sodium hydroxide, hydrochloric acid 37% and absolute ethanol were also prepared from Merck Company (Darmstadt, Germany). All reagents are analytical reagent grade. All solutions were prepared with distilled water.

### The preparation of extract

The amount of 40 g of nutmeg seeds purchased from Tabriz market (Iran). To avoid the effects of climate on the results, seeds collected form field a region were used. The seeds were mixed with 200 mL of solvent (water and acetone 1–50) and soaked at 20 °C for 24 h and stirred every two hours. After the extraction, the extract was filtered using filter paper and the filtered liquid obtained from acetone solvent was concentrated with the help of rotary evaporator at 40 °C until the complete removal of the extracted solvent and then dried. All methods on plants or plant materials were carried out in accordance with relevant guidelines in the method section.

### Analysis of extract by gas chromatography-mass spectrometry (GC–MS)

Mass chromatography (GC/MS) (Agilent 6890 model) coupled with a mass spectrometer (MS 5975) (Agilent technologies,Avondale, PA) functioning in the EI mode, equipped with a HP-5 capillary column (30 m × 0.27 mm i.d., 0.25 μm film thickness) and a flame ionization detector, were used to identify the compounds in MyR. Nitrogen gas was used as a carrier gas with a flow rate of 1 mL/min. The column temperature was programmed from 60 to 280 °C at an increasing rate of 10 °C/minute and kept at the initial and final temperatures for 2 min. Ionization method of EI with voltage ionization of 70 eV and ionization temperature of 240 °C was used. The extract was dissolved in methanol (1:100 ratio). The diluted sample of 1μL was injected into the GC column through a split injector (1:20 for 1 min) with a constant temperature of 250 °C. Compounds were identified by comparison of each mass spectra with those of pure reference compounds and confirmed using the National Institute of Standards and Technology (NIST) mass spectra library provided by the Chemstation software (version E.02.00.493)^23^.

### Synthesis of g-C3N4 and ZnO/g-C3N4 nanosheets

To prepare g-C3N4, 10 g of melamine was added to a ceramic dish and heated at 550 °C (heating speed of 5 °C/min) for four hours in an electric furnace (Azar Kiln Company, Made in Iran), after finishing the desired time. It was cooled at room temperature and the pale yellow powder was obtained^18^. Zinc acetate solution was prepared in deionized water, then it was sonicated pulse on 4 s–off 5 s for 15 min. NaOH solution was added to it under magnetic stirring at a rate of 10 ml/min and the sediments obtained were filtered, washed and dried at 100 °C for 5 h and finally powdered^49^. To synthesize the binary composite of ZnO/g-C3N4, 1 g of g-C3N4 powder was dispersed in 20 mL of methanol–water with a volume ratio of 1:1 for 30 min in the ultrasonic bath (Elma D-38687) and then 0.15 g of ZnO powder was added it and subjected to ultrasonic waves for 30 min. The suspension was stirred on the magnetic stirrer at room temperature for 12 h. The sediments were dried at 60–70 °C and then calcined at 300 °C in the electric furnace after collection by centrifuge^50^.

### Synthesis of CMC/ZnO/g-C3N4/MyR

0.8 g of CMC was dissolved in 50 mL of distilled water at 60 °C. The amount of water was stabilized in a volume of 50 ml until a completely viscous and transparent solution was obtained. The soluble temperature slowly reached room temperature. One gram of the synthesized ZnO/g-C3N4 binary composite synthesized in the previous step was added to the CMC solution and was thoroughly mixed until the gel was obtained. A rest period of 24 h was considered for the above solution to all nanoparticles were entered into the polymer matrix. For the preparation of CMC/ZnO gel (without g-C3N4), pure zinc oxide (0.15 g) was added to the same CMC volume. To prepare CMC/ZnO/g-C3N4/MyR, the extract was inserted directly into CMC at a temperature of 35 °C and a 5% w/w ratio. ZnO/g-C3N4 was added after cooling.

### Characterization

The properties of synthesized CMC/ZnO/g-C3N4 nanosheets were evaluated. Particle size was measured with the help of dynamic light distribution (DLS) using Malvern instrument UK. Measurements of nanoparticles at 25 °C and laser light exposure with wavelength of 657 nm were performed at room temperature and samples were prepared at a concentration of 0.1 mg/mL and measured 3 times and each time for 30 s. To ensure ZnO doped on the hybrid, XRD of g-C3N4 was recorded at room temperature using X' Pert Pro Panalytical diffraction, with Cu kα radiation (λ = 1.5418 Å) and working voltage of 40 kV and 40 mA. To determine the bonds produced from the FT-IR spectrum (Spectrum Two model of PerkinElmer Company) was used to determine the shape and structure of the nano-hybrids of FE-SEM analysis (FESEM device of ZEISS Company of Germany- Sigma VP model Equipped with EDS detectors, Mapping, Oxford Instruments UK company). To determine the chemical composition of X-ray photoelectron spectroscopy (XPS) (ESCALAB-MKII spectrometer; VG Co., United Kingdom (equipped with X-ray al-Kα with eV 1486.6 energy and analysis of the results was used using SPD4.1 software considering carbon peak ((1 s) C) in eV284.2 bond energy. To determine the percentage of MyR concentration at any moment, its adsorption characteristics were used. For this purpose, appropriate concentrations of MyR were prepared in lukewarm water (0.25–0.01 mg/mL) and the sample absorption rate was obtained at 326 nm (maximum absorption based on the data obtained from the scan by spectrophotometer, model Ultrospec 2000, made in Scinteck, England). Then the calibration curve was plotted and its equation was determined (Y = 0.196x + 0.0635, R2 = 0.99).

The release of MyR from the CMC/ZnO/g-C3N4/MyR was assessed in phosphate buffer saline (pH 7.4) using the dialysis bag (sigma D0530 12000 k) diffusion technique, in a thermostatically controlled water bath shaker (Memmert, GmbH; Buchenbach, Germany). An amount equivalent to 10 mg of free MyR and CMC/ZnO/g-C3N4/MyR were poured into a dialysis bag, and ends of the bag were closed, and it was immersed in a beaker containing 100 ml of release medium. The experiments were carried out at a temperature of 37 ± 1.0 °C for 48 h. The samples of 3 mL from the release medium were collected samples in time intervals and the absorbance was measured using the UV–visible spectrophotometer at 326 nm. Each withdrawn sample was replaced by an equal volume of the fresh release medium.

### Antibacterial activity

In vitro antibacterial activities of the nanosheets against *pseudomonas aeruginosa* (*P. aeruginosa*—ATCC 27853) and Staphylococcus aureus (*S. aureus*—ATCC 25923) were assessed using minimum inhibitory concentration (MIC) and minimum bactericidal concentration (MBC) as reported by others^51^. In summary, serial dilutions of nanosheets were prepared and placed in wells containing bacterial suspension (5 × 10^5^ CFU/mL). The lowest concentrations of nanosheets that prevent growth and kill bacteria were considered MIC and MBC, respectively.

### Toxicity

Cytotoxicity evaluations were conducted with the help of NIH-3T3 fibroblast cells (10^4^ cells/well) based on previous studies^52,53^. In summary, the cells were seeded in plates (10^4^ cells/well) containing different concentrations of nanosheets and incubated for 24 h. Viability was assessed with the help of optical density at 492 nm.

### In vivo studies

#### Experimental animals

The healthy BALB/c male mice (n = 90) aged 10–11 weeks with a weight of 29 ± 2 g were prepared. The animals had unlimited access to water and food. They were kept under lab conditions prior to the start of study as the conditioning period. The mice were kept under a lighting diet of 12 h. This study lasted for 12 days in accordance with the Iranian ethical guidelines for the use of animals. All the used protocols, such as study design, sample size, randomization, outcome measures, data analysis, experimental procedures, and report of results, were in agreement with the ARRIVE guidelines. The protocols were approved by the Committee on the Ethics of Animal Experiments of Veterinary Faculty and the Islamic Azad University Council on Animal Care, Tehran, Iran (IR.IAU. 1114/IAUUB.1401.09), and were in compliance with the Guide for the Care and Use of Laboratory Animals published by the US National Institutes of Health (NIH publication no.85-23, revised 1996). We declare that all methods were performed in accordance with the relevant guidelines and regulations.

#### Induction of wound model

To induce general anesthesia, Ketamine (80 mg/kg/IP) and Xylazine (15 mg/kg/IP) cocktail were used. A circular full-thickness wound was created with a diameter of 7 mm on the interscapular region of mice^52,53^. Following the induction of wound, an aliquot of 25 × 10^7^ *S. aureus* and *P. aeruginosa* was suspended in 50-μL phosphate-buffered saline (PBS) and applied on the wound region of each mouse with the help of a 200-μL pipette. Following colonization, the animals (n = 90) were randomly grouped into five groups (n = 18) and treated with 0.5 g of soft paraffin (control), standard ointment of 2% mupirocin® (Mup group) and formulations containing CMC/ZnO, CMC/ZnO/g-C3N4 and CMC/ZnO/g-C3N4/MyR and each group was exposed to 660 nm UV-light for 10 min (light power is 0.8 W cm^−2^). It should be noted that each formulation was prepared as 2% (0.4 g of synthesized material in 20 g of soft paraffin).

### Wound contraction and total bacterial count in granulation tissue

The wound contraction rate was assessed with the help of a transparent paper over the wound site and tracing it^52,53^. The total bacterial count was also measured with the help of plate count agar (Merck KGaA, Darmstadt, Germany). In summary, 0.1 g of the wound sample was crushed, minced, and diluted and cultured on plate count agar. The cultured plates were incubated at 37 °C for 48 h and the total bacterial count was calculated.

### Pathology results

On days 3, 7 and 12, the samples were prepared from the wound site, fixed, processed, paraffin wax embedded, sectioned at 5 μm, and stained with hematoxylin and eosin based on previous studies^52,53^. Edema, fibroblast, collagen and epithelization were investigated in 5 per high power fields with the help of two blinded pathologists who scored parameters in a range of 0–4 (0: Absence;1: small amount; 2: low; 3: high and 4: severe).

### Immunofluorescent staining

Immunofluorescence staining was used to evaluate the effects of nanosheets on the wound healing process and conducted as reported by others^54,55^. Following the processes of paraffin-embedding, dewaxing, rehydration and immersion into citrate buffer (pH 7.4) for 20 min, slides were washed with TBS plus 0.03% Triton X-100, blocked in 10% normal serum or with 1% BSA in TBS for 2 h at room temperature. The ratio of target protein-positive nuclei to DAPI-positive nuclei in 3 microscopic fields per group was used for quantification by ImageJ software.

The samples were investigated for CD31, COLA1 and TNF-α on day 7. The used antibodies included CD31 (pecam1-E-AB-60608-Elabscience), TNF-α (ab6671, Abcam Biotechnology, Inc), and COLA1 (sc-293182; Santa Cruz Biotechnology, Inc), Goat Anti-Mouse IgG (E-AB-1011, Elabscience Company) and Goat Anti-Rabbit IgG (E-AB-1014, Elabscience Company).

### Data analysis

The data were analyzed for normality with the help of the Kolmogorov–Smirnov test in SPSS software (version 23) and analyzed by one-way ANOVA because the data were normal. Duncan's posthoc test was used to investigate differences between groups and P < 0.05 was significant. The data for pathological were not normal and analyzed by Kruskal–Wallis.

### Ethical approval

Our Committee in Islamic Azad University of Urmia Branch (IR.IAU. 1114/IAUUB.1401.09) approved all the procedures that was in agreement with the US National Institutes of Health (NIH publication no.85-23, revised 1996).

### Supplementary Information

Supplementary Figure 1.

## Data Availability

We have not any dataset and the materials can be requested from corresponding author.

## Abbreviations

CMC: Carboxymethyl cellulose
COL1A: Collagen type 1A
EDS: Energy-dispersive X-ray spectroscopy
DLS: Dynamic light distribution
OH: Hydroxyl group
GC–MS: Gas chromatography–mass spectrometry
g-C3N4: Graphite carbon nitride
FESEM: Field emission scanning electron microscope
FT-IR: Fourier transform infrared spectroscopy
IP: Intraperitoneal injection
MBC: Minimum bactericidal concentration
MIC: Minimum inhibitory concentration
MyR: Myristica fragrans extract
Mup: Mupirocin
P. aeruginosa: *Pseudomonas aeruginosa*
S. aureus: *Staphylococcus aureus*
TNF-α: Tumor necrosis factor-α
XPS: X-ray photoelectron spectroscopy
ZnO: Zinc oxide
