# Supplementary material for: Facile fabrication of carboxymethylcellulose/ZnO/g-C3N4 containing nutmeg extract with photocatalytic performance for infected wound healing
Source: Sci Rep. 2023 Oct 31;13:18704. doi: 10.1038/s41598-023-45921-7 (PMC10618236; doi:10.1038/s41598-023-45921-7)
Supplement: Supplementary file 1 — Supplementary Figure 1. [file 41598_2023_45921_MOESM1_ESM.doc]

 Supporting Information to

**Facile fabrication of carboxymethylcellulose/ZnO/g-C3N4 containing nutmeg extract with photocatalytic performance for infected wound healing**

**Maysa Youshi, ^1^, Mohammad Reza Farahpour ^2^*, Zohreh Ghazi Tabatabaei ^3^**

*^1^* Department of Basic Sciences, Faculty of Veterinary Medicine, Urmia Branch, Islamic Azad University, Urmia, Iran.

*^2^* Department of Clinical Sciences, Faculty of Veterinary Medicine, Urmia Branch, Islamic Azad University, Urmia, Iran.

*^3^* Department of Chemistry, Ahar Branch, Islamic Azad University, Ahar, Iran.

***Correspondence to**: Mohammad-Reza Farahpour, D.V.M., D.V. Sc., Assoc. Prof. ^1^Department of Clinical Sciences, Faculty of Veterinary Medicine, Urmia Branch, Islamic Azad University, Urmia, 57159-44867, Iran. Tel: +98 4434373676. Fax: +98 443 3460980. E-mail: [mrf78s@gmail.com](mailto:mrf78s@gmail.com). ORCID ID: orcid.org/0000-0001-8631-071X


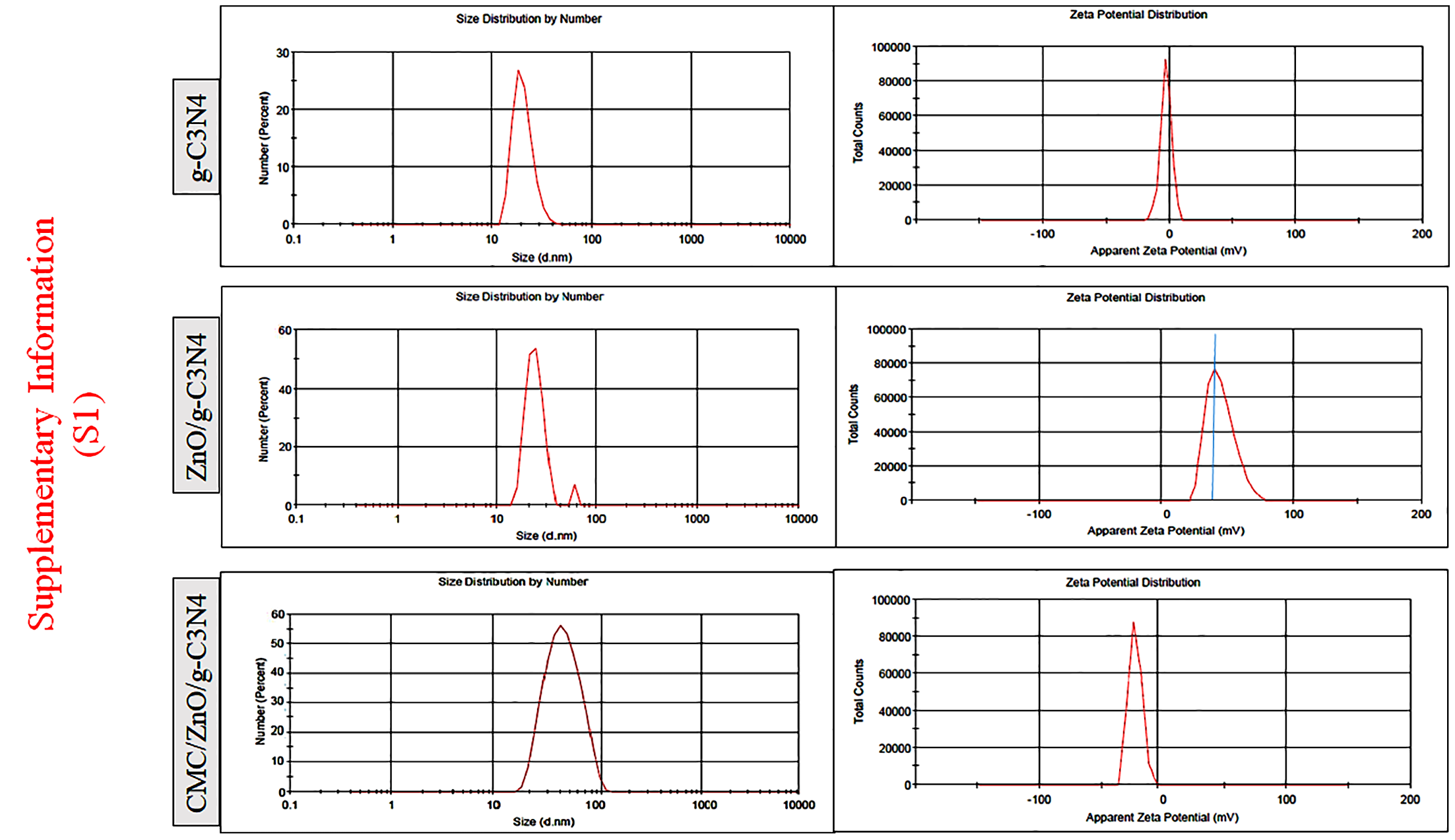


**Figure 1S:** DLS and Zeta potential of g-C3N4, ZnO/g-C3N4& CMC/ZnO/g-C3N4
